# Non-Coding RNAs at the *Gnas* and *Snrpn-Ube3a* Imprinted Gene Loci and Their Involvement in Hereditary Disorders

**DOI:** 10.3389/fgene.2012.00264

**Published:** 2012-11-26

**Authors:** Antonius Plagge

**Affiliations:** ^1^Department of Cellular and Molecular Physiology, Institute of Translational Medicine, University of LiverpoolLiverpool, UK

**Keywords:** genomic imprinting, non-coding RNA, Gnas, pseudohypoparathyroidism, Snrpn, Ube3a, Prader–Willi-syndrome, Angelman-syndrome

## Abstract

Non-coding RNAs (ncRNAs) have long been recognized at imprinted gene loci and provided early paradigms to investigate their functions and molecular mechanisms of action. The characteristic feature of imprinted genes, their monoallelic, parental-origin-dependent expression, is achieved through complex epigenetic regulation, which is modulated by ncRNAs. This minireview focuses on two imprinted gene clusters, in which changes in ncRNA expression contribute to human disorders. At the *GNAS* locus loss of *NESP* RNA can cause autosomal dominant Pseudohypoparathyroidism type 1b (AD-PHP-Ib), while at the *SNRPN-UBE3A* locus a long ncRNA and processed snoRNAs play a role in Angelman-Syndrome (AS) and Prader–Willi-Syndrome (PWS). The ncRNAs silence overlapping protein-coding transcripts in sense or anti-sense orientation through changes in histone modifications as well as DNA methylation at CpG-rich sequence motifs. Their epigenetic modulatory functions are required in early development in the pre-implantation embryo or already in the parental germ cells. However, it remains unclear whether the sequence homology-carrying ncRNA itself is required, or whether the process of its transcription through other promoters causes the silencing effect.

Imprinted gene loci provided early model systems, in which non-coding RNAs (ncRNAs) have been investigated (Barlow, 2011; Ferguson-Smith, 2011). Imprinted genes are defined as being monoallelically expressed dependent on their parental origin. During the mammalian imprinting process, epigenetic marks are established in the female or male germlines at imprinting control regions (ICRs), which results in the silencing of one parental allele in somatic cells of the offspring. A common feature of imprinted genes is their occurrence in clusters, whereby one ICR regulates the monoallelic expression of several neighboring genes, although single units of an imprinted gene and associated retrogene have also be identified (Cowley and Oakey, 2010). Imprinted gene clusters often contain ncRNAs, which are now increasingly recognized for their regulatory effects on nearby imprinted protein-coding genes. This minireview will focus on the *GNAS* and *SNRPN-UBE3A* imprinting clusters, in which disturbances of the ncRNAs are associated with human hereditary disorders. Functions of ncRNAs at other clusters have been covered in recent excellent reviews (Barlow, 2011; Ferguson-Smith, 2011; Pauler et al., 2012).

## Roles of Non-Coding RNAs at the *GNAS* Locus and Their Involvement in AD-PHP-Ib

**Figure 1:**
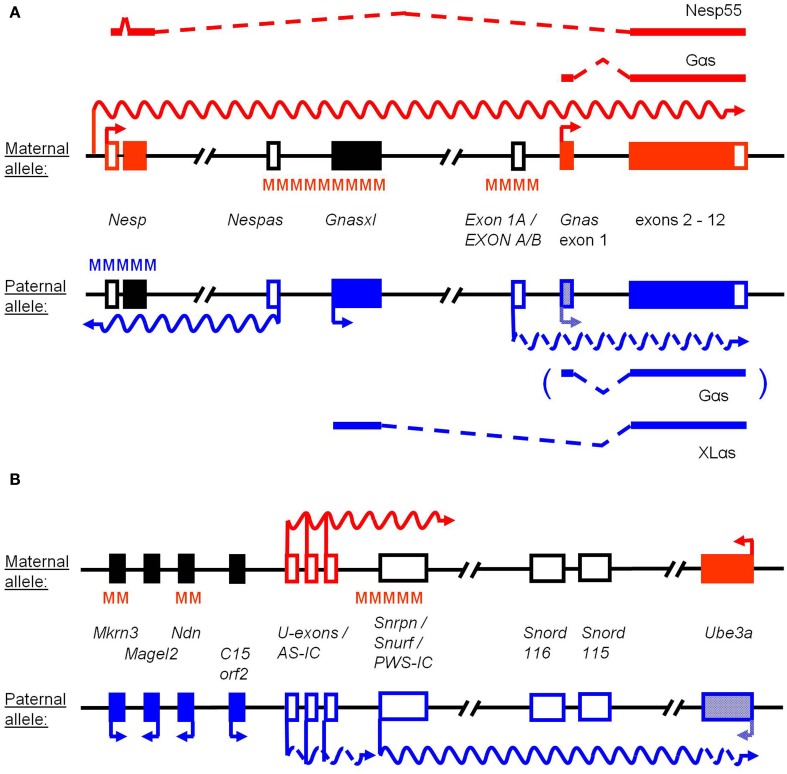
**Simplified schemes of the *Gnas* and *Snrpn-Ube3a* imprinted loci**. The features of the maternal and paternal alleles are indicated in red and blue, respectively. Genes or transcripts are named in the central part. Arrows mark transcription start sites and undulating lines ncRNAs. Open and filled boxes represent non-coding and coding exons, respectively, while black boxes indicate silenced genes. Differentially methylated regions (DMRs) of DNA are marked by MMM. **(A)** For the *Gnas* locus the alternatively spliced coding transcripts and proteins are named above and below the alleles. DMRs at *Nespas* and *Exon 1A* (*EXON A/B* in human) are established in the maternal germline, while methylation at *Nesp* occurs during early embryonic development. *Gnas* is expressed biallelically, but is silenced on the paternal allele in some tissues (hatched box), in which *Exon 1A* shows comparatively high expression levels (interrupted undulating line). *Nesp* represents a coding transcript (ORF limited to *Nesp* exon 2), but a regulatory RNA is initiated from a separate promoter in oocytes (red undulating line). *Nespas* is expressed from the unmethylated imprinting control region (ICR) of the locus. **(B)** The *Snrpn-Ube3a* imprinting cluster contains several genes (represented by single-exon boxes) that show monoallelic expression in brain. *Ube3a* is biallelically expressed in most tissues, but silenced on the paternal allele in neurons (hatched box). The *Snrpn* long ncRNA occurs in multiple, variably processed forms, including brain-specific variants that contain upstream promoters/exons (*U-exons*) and sequences overlapping with *Ube3a* (interrupted undulating lines). *Snord115* and *Snord116* represent clusters of C/D box snoRNAs, which are generated from the *Snrpn* ncRNA. The bipartite ICR of the human locus is indicated as AS-IC and PWS-IC, the latter being conserved in mouse around the main *Snrpn* start site. *C15orf2* is not present in the murine locus. Somatic DMRs at *Ndn* and *Mkrn3* are established during development. Transcription from *U-exons* occurs in the female germline in growing oocytes (red undulating line) and is involved in the establishment of methylation at the PWS-IC.

The roles of ncRNAs at the complex imprinted *Gnas* locus (Figure 1A) have become more evident through recent studies in human and mouse (Williamson et al., 2004, 2006, 2011; Bastepe et al., 2005; Liu et al., 2005a; Chotalia et al., 2009; Chillambhi et al., 2010). The locus, which is largely conserved between both species, consists of two main protein-coding transcripts (*Gnas* and *Gnasxl*) and two regulatory non-coding transcripts termed *Nespas* and *Exon 1A* (*EXON A/B* in human). A fifth transcription unit, *Nesp*, exerts a dual function within the locus through epigenetically regulating other transcripts and by encoding a protein (see below; Ischia et al., 1997; Plagge et al., 2005; Chotalia et al., 2009; Fröhlich et al., 2010). *Gnas* encodes Gαs, the α-stimulatory subunit of trimeric G-proteins, which mediates signal transduction from seven-transmembrane receptors to adenylate cyclase (Weinstein et al., 2001; Plagge et al., 2008). In some cell types, e.g., renal proximal tubules, brain subregions, thyroid, pituitary somatotroph cells among others, *Gnas* is preferentially or exclusively expressed from the maternally inherited allele (Plagge et al., 2008; Chen et al., 2009; Zazo et al., 2011). Defects in *Gnas* expression from the maternal allele can, therefore, disrupt various hormone signaling pathways, which leads to a range of disease symptoms termed “Pseudohypoparathyroidisms (PHP)” with or without “Albright’s Hereditary Osteodystrophy (AHO).” Typically, these comprise resistance to parathyroid hormone, thyroid stimulating hormone, growth hormone releasing hormone, gonadotrophins, and α-melanocyte stimulating hormone (Plagge et al., 2008; Chen et al., 2011; Mantovani, 2011). The molecular defects causing PHP/AHO can be categorized into two types: (a) mutations in the coding exons of *GNAS* or (b) epigenetic changes at the differentially methylated regions (DMRs) of the *GNAS* locus (Figure 1A). Maternally inherited coding exon mutations invariably lead to a severe combination of many PHP/AHO features, while epigenetic changes often only result in a limited spectrum of hormone resistance symptoms, mainly parathyroid hormone resistance (then also termed PHP-Ib; de Nanclares et al., 2007; Mariot et al., 2008; Kelsey, 2010; Mantovani et al., 2010; Mantovani, 2011).

The epigenetic abnormalities associated with PHP consist of changes in DNA methylation at DMRs as well as changes in ncRNA expression. Furthermore, these can occur in a familial pattern upon maternal inheritance of a mutation, i.e., autosomal dominant pseudohypoparathyroidism type 1b (AD-PHP-Ib; Bastepe et al., 2003, 2005), or sporadically, in which case the molecular cause is unknown, but most likely due to abnormalities in epigenetic regulators that act at several imprinted loci (Liu et al., 2005b; Linglart et al., 2007; Fernandez-Rebollo et al., 2011; Mantovani, 2011; Maupetit-Mehouas et al., 2011). A common epigenetic change in AD-PHP-Ib is the loss of maternal allele-specific methylation at the *EXON A/B* DMR, located a few kb upstream of *GNAS* exon 1 (Figure 1A). Interestingly, on the unmethylated paternal allele this exon acts as the start site of a ncRNA, which is transcribed across the *GNAS* promoter in the same direction (Liu et al., 2000a,b). Evidence from AD-PHP-Ib patients as well as mouse models indicates that the expression levels of the two transcripts, *EXON A/B* and *GNAS*, are oppositely regulated in *cis*. On the maternal allele methylation across *EXON A/B* inhibits expression of the ncRNA, while the downstream *GNAS* promoter drives the expression of the coding RNA (Liu et al., 2000a,b). On the paternal allele lack of methylation allows *EXON A/B* expression, while the *GNAS* promoter is suppressed in at least some cell types, i.e., *GNAS* expression becomes imprinted, for example in renal proximal tubules. In AD-PHP-Ib patients two types of deletion mutations upstream of *GNAS* result in loss of methylation at the *EXON A/B* DMR. One of these comprises a 1.3 kb region in the neighboring gene *STX16* ∼220 kb upstream of *GNAS* (Bastepe et al., 2003; Linglart et al., 2005), and the other deletion affects the most 5′ region within the complex *GNAS* locus (i.e., *NESP* and *NESPAS* exons; Bastepe et al., 2005; Chillambhi et al., 2010; Richard et al., 2012). For the latter type of deletion it has been shown that the loss of methylation at *EXON A/B* is not only associated with a loss of *GNAS* expression, but also with an increase in the levels of the non-coding *EXON A/B* RNA (Bastepe et al., 2005; Fröhlich et al., 2010). This raises the question of whether the *EXON A/B* RNA, or the process of its transcription, regulates *GNAS* expression. Further insights have been provided recently through a mouse model, in which the *Exon 1A* RNA has been truncated by insertion of a polyadenylation cassette (Eaton et al., 2012). Paternal inheritance of this allele results in early termination of *Exon 1A* transcription, thus avoiding extension across the downstream *Gnas* promoter region. DNA methylation across the *Exon 1A* DMR is not changed in this model. However, the expression of *Gnas* becomes up-regulated in tissues where it is normally imprinted and repressed (and in which normally comparatively high levels of *Exon 1A* RNA are found; Eaton et al., 2012). These observations favor a mechanism, whereby the non-coding *Exon 1A* RNA, or the process of its transcription through the *Gnas* promoter, inhibits the expression of the latter (transcriptional interference), although alternative mechanisms (e.g., impairment of a silencer element for *Gnas* in the *Exon 1A* region) cannot be excluded at this stage (Liu et al., 2000a; Eaton et al., 2012).

In contrast to the *Exon 1A* transcript, the *Nespas* ncRNA functions in an anti-sense orientation, to counter-regulate, and silence the transcription of the Nesp55 coding RNA (Hayward and Bonthron, 2000; Wroe et al., 2000; Williamson et al., 2011). *Nespas* is expressed from a separate promoter, which is located upstream of *Exon 1A* and *Gnas* in the imprinting control center (ICR) of the locus (Figure 1A; Williamson et al., 2006). It is transcribed in the opposite direction of *Exon 1A* and *Gnas* and from the paternal allele only. Its promoter is methylated on the maternal allele (Hayward and Bonthron, 2000; Wroe et al., 2000; Williamson et al., 2002). The role of the *Nespas* ncRNA was investigated using a polyadenylation cassette again, to terminate the transcript a short distance downstream of its initiation site (Williamson et al., 2011). The authors showed that the truncated RNA had lost its anti-sense silencing function for *Nesp* on the paternal allele. The DNA methylation at the *Nesp* promoter on the paternal allele, which is normally established during early embryogenesis, was lost and *Nesp* became biallelically expressed. A second, hypomorphic *Nespas* mutant (>90% loss of RNA levels, but no truncation) revealed further insights into the mechanisms, by which the ncRNA silences *Nesp* (Williamson et al., 2011). In this model DNA methylation on the paternal allele of the *Nesp* DMR was also lost, but, in contrast to the truncation model, expression of *Nesp* RNA was only partially up-regulated. The reason for the different levels of induction of *Nesp* transcription in the *Nespas* truncation vs. hypomorph model was found to be related to histone modifications. While the activating histone mark H3K4me3 is usually depleted at the *Nesp* promoter on the paternal allele, increased levels were found in both *Nespas* mutants, in line with *Nesp* transcriptional activation. Moreover, the H3K4me3 levels correlated with the degree of transcriptional activation of *Nesp* as these were higher in the *Nespas* truncation model (Williamson et al., 2011). Thus, under normal conditions *Nespas* anti-sense transcription through the *Nesp* promoter results in low H3K4me3 levels and suppression of *Nesp*. Furthermore, the increased H3K4me3 levels in both *Nespas* mutants are a likely cause for the lack of DNA methylation at the *Nesp* DMR, since this histone modification is incompatible with the action of the DNA methyltransferases Dnmt3a and 3L (Ooi et al., 2007; Zhang et al., 2010). Other changes in histone marks of both *Nespas* mouse models included a depletion of repressive H3K9me3 downstream of the *Nesp* start site and a depletion of the transcription elongation mark H3K36me3 upstream of *Nesp*, which is consistent with up-regulation of *Nesp* and loss of *Nespas* transcription, respectively, on the paternal allele (Williamson et al., 2011). Overall, these findings indicate a major role for the non-coding anti-sense RNA *Nespas* (or for its transcription process) in mediating the silencing and stable suppression of *Nesp* in *cis* through low levels of H3K4me3 and, consequently, DNA methylation via Dnmt3a/L. The attraction of histone modifying enzymes, e.g., a histone demethylase, through the ncRNA appears to be a likely molecular mechanism. Whether the changes of *Nespas* and *Nesp* expression on the paternal allele of these mouse models further affect the downstream *Exon 1A* and *Gnas* transcription units, potentially leading to loss of imprinting of *Gnas* as described in the *Nespas* ICR deletion mice, remains to be analyzed (Williamson et al., 2006, 2011).

The theme of epigenetic regulation through RNAs (or through the process of their transcription across other promoters) extends to a third RNA of the *Gnas* locus, namely the protein-coding *Nesp* (Ischia et al., 1997; Plagge et al., 2005; Chotalia et al., 2009). Dual functions as a coding and ncRNA are unusual, but have recently been described in several cases (Cooper et al., 2011; Kageyama et al., 2011). The regulatory role of *Nesp* occurs in the female germline in growing oocytes at postnatal stages when maternal DNA methylation imprints are established at the *Nespas* ICR and *Exon 1A* germline DMR (Chotalia et al., 2009). At this stage, an oocyte-specific promoter drives the transcription of *Nesp*, which extends through all other promoters and exons of the *Gnas* locus (Figure 1A). When a polyadenylation/transcriptional termination cassette was used to truncate the *Nesp* RNA shortly after its single-exon open reading frame on the maternal allele, the DNA methylation marks at the *Nespas* ICR and *Exon 1A* germline DMR failed to be established (Chotalia et al., 2009). Offspring that inherited this truncation maternally showed epigenetic and transcriptional patterns on the maternal chromosome in somatic tissues that resembled those normally found on the paternal chromosome, i.e., loss of DNA methylation was associated with expression of the *Nespas*, *Gnasxl*, and *Exon 1A* transcripts. Consequently, transcription of *Nesp* and *Gnas* (in imprinted tissues) became silenced and the somatic *Nesp* DMR methylated on the maternal chromosome (Chotalia et al., 2009). The consequences of the *Nesp* truncation are, therefore, very similar to the *NESP* deletion mutations found in patients with AD-PHP-Ib described above (Bastepe et al., 2005; Chillambhi et al., 2010; Richard et al., 2012) and to the mouse *Nesp* deletion model (Fröhlich et al., 2010). The comparison of these various mutants emphasizes the importance of the process of transcription (or generation of overlapping RNA) for establishing epigenetic modifications, which then last throughout development. Regarding details of the molecular mechanisms involved, several possibilities have been discussed and a crucial role of histone modifications/modifying enzymes and their interaction with DNA methyltransferase and/or RNA Polymerase complexes appear most likely (Smallwood and Kelsey, 2012).

## Roles of the Long ncRNA *SNRPN* at the PWS/AS Locus

Another cluster of imprinted genes that is regulated by a large ∼0.5–1.0 Mb ncRNA, termed *SNRPN*, is located on human chromosome 15q11-13 and is largely conserved on mouse chromosome 7 (Figure 1B; Landers et al., 2004; Buiting, 2010). In humans, the locus is associated with the neurogenetic imprinting disorders Prader–Willi-Syndrome (PWS) and Angelman-Syndrome (AS; Buiting, 2010; Mabb et al., 2011). AS is caused by loss of expression of *UBE3A* from the maternal allele. *UBE3A* encodes an E3 ubiquitin ligase, which is only imprinted in brain and biallelically expressed in other tissues. Among other symptoms, the disorder is characterized by developmental delays, intellectual disability, and behavioral abnormalities (happy demeanor; Mabb et al., 2011). By contrast, the symptoms of PWS, which include neonatal hypotonia, feeding difficulties, and growth retardation followed from early childhood onward by hyperphagia, severe obesity, hypogonadism, and behavioral abnormalities, is caused by loss of expression of several paternally expressed genes of the locus (Buiting, 2010). Mouse models and human genetic studies indicate that the genes *NECDIN* (*NDN*), *MAGEL2*, and parts of the long ncRNA (lncRNA) *SNRPN* contribute to this complex disorder (Figure 1B; Gerard et al., 1999; Muscatelli et al., 2000; Kozlov et al., 2007; Sahoo et al., 2008; de Smith et al., 2009; Duker et al., 2010). The lncRNA *SNRPN* can be processed and spliced in many ways and is expressed in most tissues. Some of its transcript variants are neuron-specific, initiated at separate upstream exons (*U-exons*), and overlap anti-sense with *Ube3a* (then also termed *UBE3A-ATS*; Bressler et al., 2001; Runte et al., 2001; Yamasaki et al., 2003; Landers et al., 2004; Le Meur et al., 2005). The *U-exon/SNRPN/UBE3A-ATS* lncRNA has a crucial role in silencing *UBE3A* on the paternal allele in neural tissue. The *SNRPN* main transcriptional start site is located within the PWS-IC, a 4.3 kb (or 6.0 kb in mouse) region, which remains unmethylated on the paternal allele and is thought to contain an activator function for all paternally expressed genes, including those located upstream of the lncRNA (Figure 1B; Buiting, 2010; DuBose et al., 2011). This function of the PWS-IC, to guarantee paternal gene expression, appears to be required at a critical time point during pre-implantation development, but not anymore at later stages, e.g., in neuronal precursor cells (DuBose et al., 2012; Rabinovitz et al., 2012). It remains to be clarified whether transcription of the *Snrpn* lncRNA in early embryos might attract histone modifications, which consequently lead to the permanent setting of all epigenetic marks of the paternal allele (Makedonski et al., 2005; Mabb et al., 2011), resulting not only in activation of paternally expressed genes, but also silencing of *Ube3a* in brain. Alternatively, *Ube3a* might be silenced directly on the level of transcriptional processes, since no differential DNA methylation occurs at this gene (Mabb et al., 2011). A truncated *Ube3a-ats* RNA that still contains the *Snord* RNA clusters (Figure 1B; see below), but does not overlap with *Ube3a* anymore, lost its silencing function on the latter in a cell culture model, indicating that the lncRNA needs to contain anti-sense sequences to *Ube3a* or that the process of transcription through the coding gene is required (Meng et al., 2012).

The *SNRPN* lncRNA has a unique additional feature as it contains two large clusters of small nucleolar RNAs (C/D box snoRNAs), termed *SNORD115* and *SNORD116*, which are predominantly expressed in the brain and, in the case of *SNORD116*, contribute to many of the PWS symptoms (Sahoo et al., 2008; de Smith et al., 2009; Duker et al., 2010; Bortolin-Cavaille and Cavaille, 2012). The evidence from human PWS patients carrying microdeletions of the *SNORD116* cluster has been confirmed in mice with targeted deletion of the corresponding elements, although the murine phenotype is largely restricted to the neonatal hypotonia and growth retardation of PWS and does not reproduce the obesity and infertility found in adult PWS patients (Skryabin et al., 2007; Ding et al., 2008). The *Snord115* and *116* clusters are processed from *Snrpn* and show the typical features of C/D box snoRNAs. However, their precise molecular functions remain elusive as they do not show the usual anti-sense sequence characteristics to ribosomal or spliceosomal RNAs (Bortolin-Cavaille and Cavaille, 2012).

In humans, the expression of the *SNRPN* lncRNA from the PWS-IC is itself regulated by a second element, which is located ∼30 kb upstream and termed AS-IC (Figure 1B; Buiting, 2010; Mabb et al., 2011). The AS-IC mediates methylation of the PWS-IC on the maternal chromosome and silencing of *SNRPN*, which in turn allows *UBE3A* expression. The AS-IC element was shown to bind a number of proteins, including transcription factors, but is not conserved as an orthologous element in mice (Kaufman et al., 2009). However, details of the PWS-IC imprinting mechanism in mice have recently emerged, showing that transcription through the *Snrpn* promoter from upstream exons (*U-exons*) in oocytes brings about the DNA methylation and silencing of *Snrpn* exon 1 on the maternal allele (Mapendano et al., 2006; Smith et al., 2011). In a BAC transgenic model a subset of *U-exons* (contained within a ∼100 kb region 5′ of *Snrpn* exon 1) caused transcription through the PWS-IC/*Snrpn* promoter in growing oocytes. This transcriptional activity led to DNA methylation at the PWS-IC in *cis* and silencing of the *Snrpn* lncRNA expression, i.e., establishment of the maternal imprint mark. There is also evidence for the presence of a corresponding *U-exon* in the human AS-IC region, which might allow for a similar mechanism of transcription/RNA mediated epigenetic modification (Farber et al., 1999). As with the *Gnas* locus, it remains unclear whether the RNA is required or whether the process of transcription through the *Snrpn* promoter suffices, to establish the epigenetic/imprinting mark at the PWS-IC (Chotalia et al., 2009; Smith et al., 2011). However, the functional similarities in oocytes between the *U-exons* of *Snrpn* and *Nesp* of the *Gnas* locus are evident, and it has been shown recently that transcription is a common feature associated with the establishment of DNA methylation in oocytes (Smallwood et al., 2011).

## Conflict of Interest Statement

The author declares that the research was conducted in the absence of any commercial or financial relationships that could be construed as a potential conflict of interest.
